# Characteristics of *Mycoplasma hyopneumoniae* Strain ES-2 Isolated From Chinese Native Black Pig Lungs

**DOI:** 10.3389/fvets.2022.883416

**Published:** 2022-06-30

**Authors:** Bingbing Zong, Yongwei Zhu, Manli Liu, Xiangru Wang, Huanchun Chen, Yanyan Zhang, Chen Tan

**Affiliations:** ^1^Hubei Key Laboratory of Animal Nutrition and Feed Science, Wuhan Polytechnic University, Wuhan, China; ^2^State Key Laboratory of Agricultural Microbiology, College of Veterinary Medicine, Huazhong Agricultural University, Wuhan, China; ^3^Hubei Biopesticide Engineering Research Centre, Hubei Academy of Agricultural Sciences, Wuhan, China; ^4^Key Laboratory of Preventive Veterinary Medicine in Hubei Province, The Cooperative Innovation Center for Sustainable Pig Production, Wuhan, China; ^5^Key Laboratory of Development of Veterinary Diagnostic Products, Ministry of Agriculture of the People's Republic of China, Wuhan, China; ^6^International Research Center for Animal Disease, Ministry of Science and Technology of the People's Republic of China, Wuhan, China

**Keywords:** *Mycoplasma hyopneumoniae*, Chinese native black pig, swine enzootic pneumonia, ES-2 strain, characteristics

## Abstract

*Mycoplasma hyopneumoniae* is the primary pathogen of swine enzootic pneumonia and causes great economic losses to the swine industry worldwide. In China, *M. hyopneumoniae* seriously hinders the healthy development of the native black pigs. To prevent and treat porcine respiratory disease caused by *M. hyopneumoniae*, the characteristics of *M. hyopneumoniae* strain ES-2 isolated from Chinese native black pig lungs with gross lesions at post-mortem were studied for the first time in this study. Strain ES-2 cell was round or oval cells and most sensitive to kanamycin. The diameters of most strain ES-2 cells ranged from 0.4 to 1.0 μm with maximum viability of 10^10^ CCU/ml. Experimental challenge of animals with strain ES-2 showed respiratory disease could be reproduced, with pneumonic lung lesions evident. Comparative genomics analysis identified that 2 genes are specific to pathogenic *M. hyopneumoniae* strains, which may be predicted to be a molecular marker. These findings suggest that the study on the characteristics of *M. hyopneumoniae* strain ES-2 will guide the rapid and accurate drug use in the clinic, and develop a theoretical foundation for accurately diagnosing and treating the infection caused by pathogenic *M. hyopneumoniae*.

## Introduction

*Mycoplasma hyopneumoniae* (*M. hyopneumoniae*) is a host-specific microorganism that only infects swine (1, 2). *M. hyopneumoniae* does not have a cell wall, and its genome consists of 893–920 kilobase pairs which encode only a few biosynthetic pathways (3, 4). Due to the limited metabolism, *M. hyopneumoniae* needs to obtain essential metabolites from the host (5). In addition, *M. hyopneumoniae* is very susceptible to the change in environmental conditions since it lacks a cell wall, thus it is difficult to cultivate *M. hyopneumoniae* in the medium without cells (6). Although the cultivation of *M. hyopneumoniae in vitro* has been improved since the 1970s (7), the successful isolation of *M. hyopneumoniae* is still complicated (8). *M. hyopneumoniae* mainly infects pig lung tissue, once *M. hyopneumoniae* invades and colonizes the respiratory tract of pigs, the pathogen will persist in the host (9, 10). *M. hyopneumoniae* first adheres to, and then colonizes the ciliated epithelial cells of the respiratory tract (1), resulting in ciliostasis, clumping, and finally loss (11). Once the ciliary activity of the respiratory tract is impaired, mucosal immunity and clearance is damaged (12). In addition, *M. hyopneumoniae* is capable of modulating both innate and adaptive immune systems (13), leading to the damage of the host defense system and the chronic colonization of this pathogen in the respiratory tract (4). Clinically, *M. hyopneumoniae* is able to infect suckling piglets, weaned piglets, and fattening pigs. To date, *M. hyopneumoniae* has mostly been detected in intensive swine production farms and herds all over the world (14, 15). Therefore, *M. hyopneumoniae*, as a widespread respiratory pathogen, is the primary etiological agent responsible for enzootic pneumonia (EP) which is a chronic respiratory disease causing huge economic losses to the pig industry world (16).

About 100 years ago, the chronic and prevalent EP were described (17). In 1963, *M. hyopneumoniae* strain J was isolated (18). Subsequently, *M. hyopneumoniae* was identified as the causative agent of EP in 1965 (7). In 1975, Friis et al. (7) reported that the Friis medium can be used to isolate and cultivate *M. hyopneumoniae*. Although it is very complicated and time-consuming to isolate *M. hyopneumoniae* from lung tissue of pigs, several strains of *M. hyopneumoniae* J (GenBank assembly accession: GCA_000008205.1), *M. hyopneumoniae* 7422 (GenBank assembly accession: GCA_000427215.1), *M. hyopneumoniae* 7448 (GenBank assembly accession: GCA_000008225.1), *M. hyopneumoniae* 168 (GenBank assembly accession: GCA_000183185.1), *M. hyopneumoniae* 232 (GenBank assembly accession: GCA_000008405.1), *M. hyopneumoniae* KM014 (GenBank assembly accession: GCA_002257505.1), *M. hyopneumoniae* TB1(GenBank assembly accession: GCA_002213485.1), and *M. hyopneumoniae* 11 (GenBank assembly accession: GCA_002193015.1) have been reported (8, 19–24). And their genomes are available in GenBank. Enshi black pigs, a typical Chinese native black breed, are best known for cold-wet tolerance and their fat storage ability, which is mainly raised in mountainous regions with an average altitude of 800 m above sea level in southwest China. However, they suffer from swine EP for a long time. So far, *M. hyopneumoniae* which causes Enshi black pig EP has not been studied. To effectively prevent and treat the EP in Enshi black pigs, *M. hyopneumoniae* from the lungs of Enshi black pigs will be isolated and cultured in this study.

In addition, with the rapid development of the pig industry, porcine respiratory diseases have become more and more complicated (25–27). In clinical cases, respiratory disease caused by only one pathogen is very rare. In most cases, the respiratory disease is complicated, which is caused by multiple pathogens, namely, *M. hyopneumoniae*, porcine circovirus type 2 (PCV-2), porcine reproductive and respiratory syndrome virus (PRRSV), and so on (28, 29). Of which *M. hyopneumoniae* is one of the main pathogens (30). To effectively prevent and control complex porcine respiratory diseases, the characteristics of *M. hyopneumoniae* were studied by many countries. Moreover, a variety of methods that can accurately detect *M. hyopneumoniae* in pig lung lesions were developed, and the sensitivity of the isolates to different antibiotics was determined (25–27, 29). Simultaneously, as more and more *M. hyopneumoniae* genomes were available, many genes that play important roles in the growth or pathogenicity of *M. hyopneumoniae* were identified by comparative proteomics, transcriptomic, and comparative genomics analyses (23, 24, 31–34). These findings provide a lot of reliable theoretical basis for the local treatment and prevention of *M. hyopneumoniae* infection. Therefore, the characteristics of the *M. hyopneumoniae* strain isolated from Enshi black pig lungs will be studied, which will lay a theoretical foundation for the development of treatment for infections caused by *M. hyopneumoniae*.

In this study, *M. hyopneumoniae* strain ES-2 was isolated from Chinese native pig lungs with gross lesions at post-mortem and determined the growth characteristics and the pathogenicity of isolates. The susceptibility of *M. hyopneumoniae* strain ES-2 to different antibiotics was determined. Based on the comparative genomics analyses, the genes which are present in pathogenic *M. hyopneumoniae* strains and absent in nonpathogenic *M. hyopneumoniae* strains will be identified. This study will provide a theoretical foundation to effectively prevent the EP.

## Materials and Methods

### Strains and Culture Conditions

*Mycoplasma hyopneumoniae* (*M. hyopneumoniae*) ES-2 strain is a China field isolate from the lung with gross lesions of Enshi Black pig. And the lung was from the slaughterhouse. *M. hyopneumoniae* strain ES-2 was isolated by using Friis liquid medium modified Friis liquid medium and modified Friis solid medium as previously described (19).

### Method of Isolating *M. hyopneumoniae* Strain ES-2 and Its Sensitivity to Kanamycin

To improve the isolation rate of *M. hyopneumoniae*, the lung is required to be harvested from 3–4-month-old, chronic coughing, and skinny pigs with obvious lesions of *M. hyopneumoniae* pneumonia in the apical and cardiac lobes of the lung. The lungs harvested from the diseased pig were immediately put into the clean bench. A total of 9 lung tissue samples from the different sites of the same lung were placed in a centrifuge tube with sterile phosphate-buffered saline (PBS), and then cut the lung tissue in the centrifuge tube into small pieces with scissors and grind it with a tissue grinder. Subsequently, the supernatants were collected by centrifugation at 5,000 rpm for 10 min, and bacteria in the supernatants were removed by 0.45 μm filtration. The supernatants were transferred into a fresh Friis medium. The mixtures were incubated in 5% CO_2_ at 37°C for 6 days. On day 6 of culture, *M. hyopneumoniae* in Friis medium was identified by PCR amplifying the highly conserved 16S rRNA region from *M. hyopneumoniae* (19). The samples identified correctly by PCR were continued to subculture. If PCR identification of the culture at passage 4 was still correct, the positive samples were placed on the modified Friis solid medium. After culture for 9 days, a single colony on the solid medium was cultured in the modified Friis liquid medium. After three times of purification on the modified Friis solid medium, the purified colony was identified by PCR and sequencing.

Minimal inhibitory concentrations (MICs) of kanamycin for planktonic cultures of *M. hyopneumoniae* ES-2 were determined using a broth microdilution method previously described (35).

### Scanning Electron Microscopy (SEM) and Transmission Electron Microscopy (TEM)

For the purpose of the visualization of *M. hyopneumoniae* ES-2 strain, scanning electron microscopy (SEM) and transmission electron microscopy (TEM) were performed as previously described with some modifications (36). The insoluble impurities in the fresh-modified Friis medium were removed by centrifugation at 10,000 rpm for 20 min and then centrifuged fresh-modified Friis medium was used to culture the ES-2 strain. Two days after culture, 100 ml of cultured ES-2 was centrifuged at 12,000 rpm for 15 min at 4°C, and the pellets were washed three times with PBS. The pellets were fixed with 2.5% glutaraldehyde in PBS at 4°C for 2 h. And then samples were sent to Servicebio for further processing. ES-2 was visualized using an HT7700 TEM and a SU8010 SEM. The adobe photoshop CC was used to determine the diameter of ES-2.

### Viability Determination of Strain ES-2 in Liquid Medium and on Solid Medium

More than 20 continuous passages of *M. hyopneumoniae* ES-2 were performed. And the colonies of the ES-2 strain were cultured to confirm that there are no other microorganisms except *M. hyopneumoniae* ES-2 strain in the medium. And then ES-2 was inoculated into fresh-modified Friis medium. The mixture was cultured in 5% CO_2_ at 37°C for 6 days. Meanwhile, the growth of ES-2 was examined every 12 h, between 0 and 144 h with the metabolic activity recorded by the color-changing units (CCU) which was indicated by the phenol red as previously described (37).

To observe the growth characteristic of ES-2 strain on a solid culture medium, the modified Friis solid medium was prepared as previously described (19). ES-2 at the exponential growth phase with five-fold serial dilutions in fresh-modified Friis medium was spotted (200 ml) onto the modified Friis solid medium. After ES-2 was cultured in 5% CO_2_ at 37°C for 9 days, ES-2 strain colonies were observed by Inverted Microscope (Olympus, Japan).

### Animal Experimental Design

Three-week-old piglets were purchased from a commercial herd. Before *M. hyopneumoniae* inoculation, blood samples were collected from each piglet. Anti-*M. hyopneumoniae* IgG antibody in sera, PCV-2 and PRRSV in blood were separately detected by the test kit (IDEXXCo.) and PCR was used to confirm that no piglets were exposed to *M. hyopneumoniae*, PCV-2, or PRRSV. On the 28th day, 4-week-old pigs were weighed and randomly allocated to two different groups. Different groups with 5 pigs in each group were housed in different rooms to avoid the spread of *M. hyopneumoniae* between groups. All pigs were inoculated and then euthanized as described previously (38) with minor modifications. Experimental group was inoculated intratracheally with 7 ml inoculum containing 7×10^7^ CCU of *M. hyopneumoniae* ES-2 strain. The control group was inoculated with 7 ml modified Friis liquid medium without ES-2.

On day 38 after infection, all pigs were euthanized and necropsied as previously described (38). For histopathological analysis, the parts of the lungs were fixed in 4% paraformaldehyde. And then, the fixed lungs were processed, stained with H&E, and visualized under a light microscope. To observe the ciliated epithelial cells and microvilli of the tracheal surface of the infected lungs, the lung bronchi were gently washed three times with 0.1 M PBS, and then were immediately put into fixative (G1102, Servicebio) at 4°C for 2–4 h for TEM. The remaining lungs were stored at −20°C, which were used to identify *M. hyopneumoniae* by PCR and isolate the ES-2 strain.

### Genome Sequencing, Assembly, and Annotation

After recovery of the frozen *M. hyopneumoniae* ES-2 strain, the liquid culture with ES-2 strain was placed on a modified Friis solid medium. After culture for 9 days, the colony of *M. hyopneumoniae* ES-2 strain was cultured in 150 ml modified Friis liquid medium at 37°C. And then the *M. hyopneumoniae* ES-2 strain was harvested by centrifugation at 14,000 g for 15 min. Afterward, the DNA of the ES-2 strain was extracted by using a TIANamp Genomic DNA Kit (Q5502, Tiangen, China). The complete genome of the *M. hyopneumoniae* ES-2 strain was sequenced using the PacBio method. The raw reads (approximately 906,783,884 bp polymerase read bases) were obtained by the third-generation sequencing. Polymerase read quality was 0.876. The reads were assembled *de novo* using the HGAP2 protocol on the SMRT Portal v2.3.0 (39). A circular chromosome was obtained with 751× average base coverage. The annotation and analysis of the sequences of the genome were performed using Glimmer3 v3.02 (40) with the default parameters.

### Phylogenomic Analyses

The complete sequences of a total of 162 *mycoplasma* strains were collected from the genome assembly database. A phylogenetic tree including 59 human isolates, 35 bovine isolates, 17 porcine isolates, 18 chicken isolates, 13 sheep isolates, and 20 other isolates (unknown host) was generated based on CDS existence information in 162 mycoplasma strains using amino acid multiple sequence alignment in Roary v3.11.2 (http://sanger-pathogens.github.io/Roary/) with default parameters (41). To analyze the genes which are present in pathogenic *M. hyopneumoniae* strains and absent in nonpathogenic *M. hyopneumoniae* strains, BlastP with a similarity cutoff (0.75, 0.80, 0.85, 0.90, 0.95, and 0.99) (42) was performed to distinguish homologous protein-coding regions from nonhomologous ones. These diagrams were developed by VENNY.

## Results

### The Isolation and Identification of *M. hyopneumoniae* Strain ES-2

**Figure 1:**
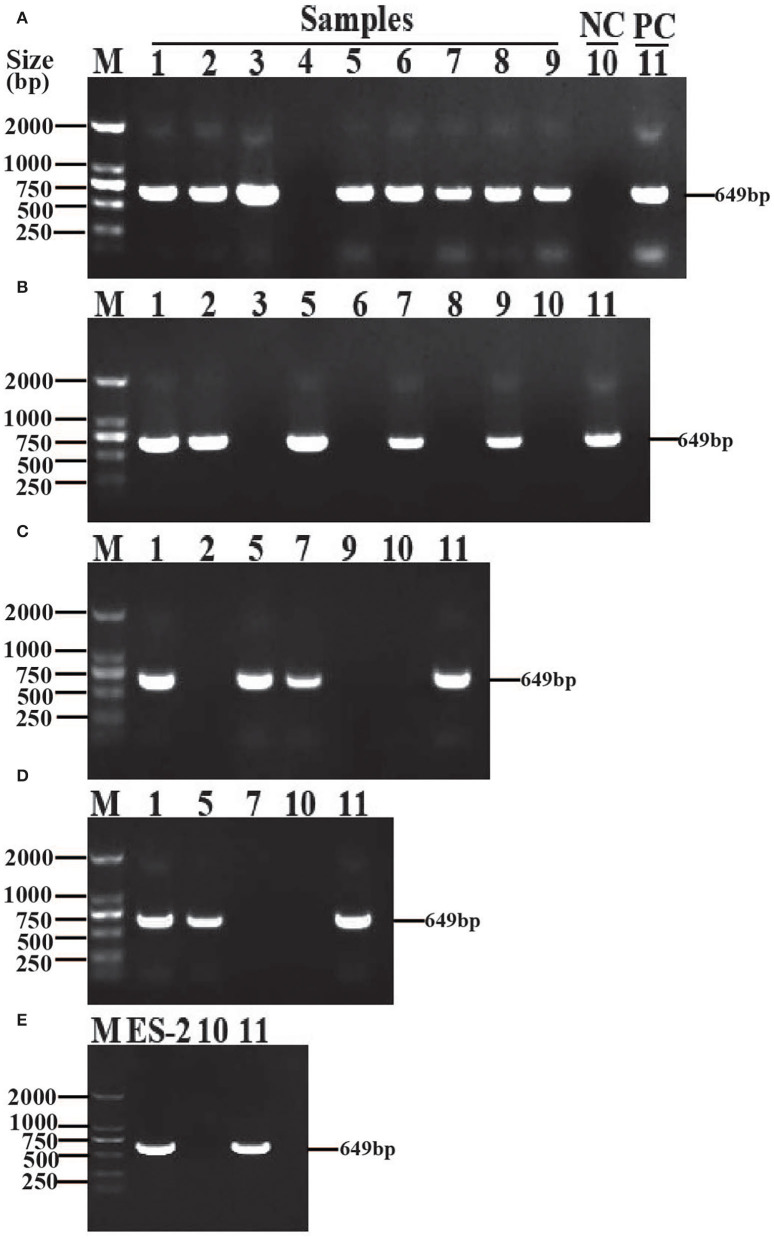
Isolation and identification of *M. hyopneumoniae* strain ES-2. The samples (1–9) were harvested from different parts of the same lung with gross lesions of EP, *M. hyopneumoniae* strain ES-2 was isolated by *in vitro* continuous passage and was confirmed by amplifying the highly conserved 16S rRNA region through PCR with the forward primer 5′-GAGCCTTCAAGCTTCACCAAG A-3′and the reverse primer 5′-TGTGTTAGTGACTTTTGCCACC-3′. **(A)** The first generation. **(B)** The second generation. **(C)** The third generation. **(D)** The fourth generation. **(E)** The twentieth generation. M, marker; NC, negative control; PC, positive control.

After the first generation was cultured for a week, 8 out of 9 samples were observed to be positive by PCR (Figure 1A). The 8 positive samples were separately transferred into a fresh Friis liquid medium (1:4). After one week's culture, 5 out of 8 samples were found to be positive by PCR (Figure 1B). These 5 positive samples were separately transferred into fresh liquid Friis medium (1:4). After one week's culture, 3 out of 5 samples were detected to be positive by PCR (Figure 1C). In this continuous passage culture, 2 samples of the fourth generation remained positive by PCR (Figure 1D). Subsequently, the serial passage culture of these 2 positive samples was performed in the modified Friis liquid medium. After 20 passage cultures, *M. hyopneumoniae* was still identified by PCR (Figure 1E).

To get pure *M. hyopneumoniae* strain, the liquid cultures were placed on the modified Friis solid medium. And then a single colony was cultured in the modified Friis liquid medium. After three times of purification, sequencing results showed that the microorganism purified in liquid medium is *M. hyopneumoniae* strain (GenBank accession number GCA_004768725.1). Ultimately, a field *M. hyopneumoniae* strain was successfully isolated and purified from the lung of Enshi black pigs suffering from typical swine mycoplasma pneumonia. And we named this newly isolated strain *M. hyopneumoniae* strain ES-2. Meanwhile, this study found that the minimum inhibitory concentration of *M. hyopneumoniae* ES-2 strain to kanamycin was 2 μg/ml.

### Colonies of *M. hyopneumoniae* Strain ES-2 on Solid Media and Morphology of ES-2 Cells Visualized by Electron Microscope

**Figure 2:**
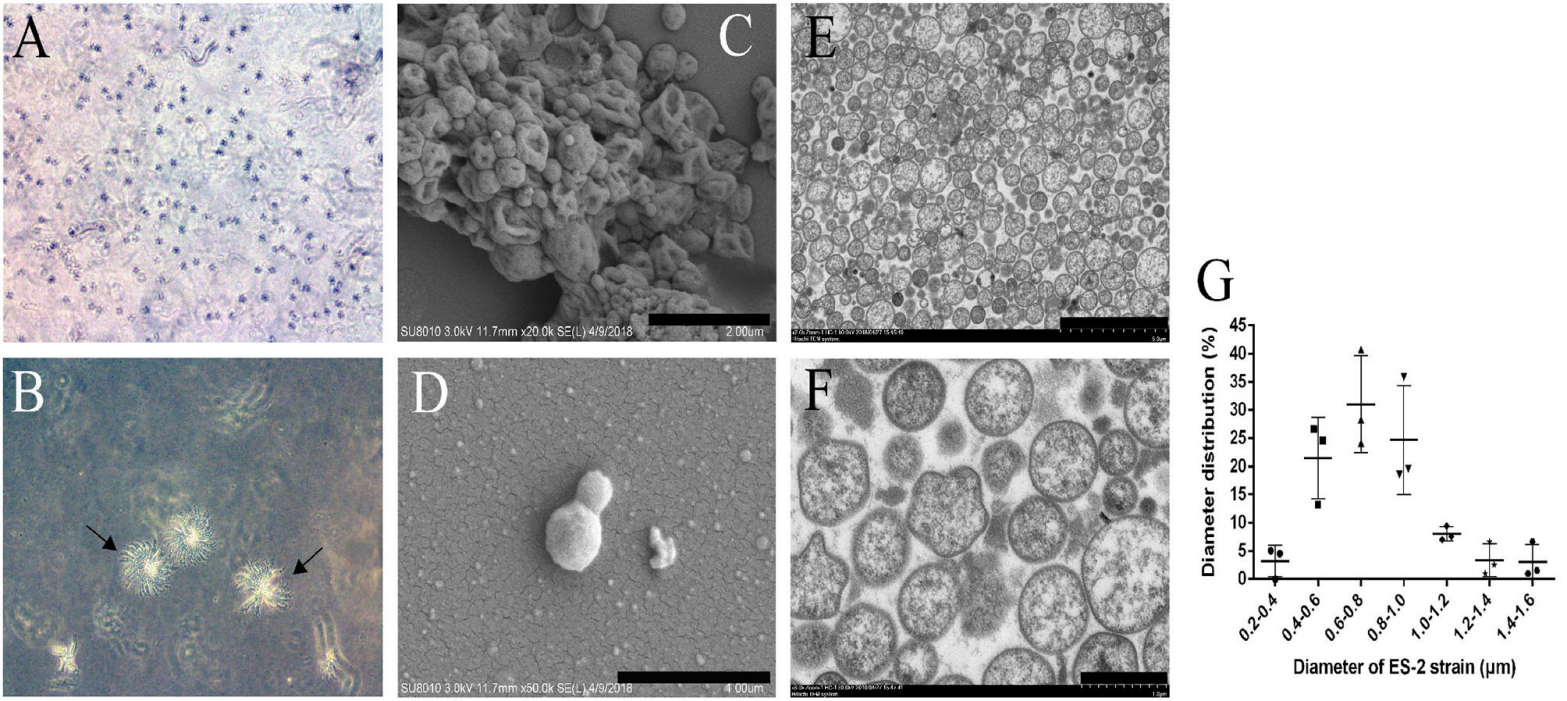
Colony of *M. hyopneumoniae* strain ES-2 on modified Friis solid media and the ES-2 cell morphology viewed under the electron microscope. A, B The colony of ES-2 strain were viewed by the inverted microscope, **(A)** 5× and **(B)** 10×. **(C,D)** The morphology of strain ES-2 was viewed by scanning electron microscope. **(C)** Bars = 2.0 μm, **(D)** Bars = 1.0 μm. **(E,F)** The cytoplasmic structure of strain ES-2 was viewed by transmission electron microscope. **(E)** Bars = 5.0 μm, **(F)** Bars = 1.0 μm. **(G)** The diameter of the ES-2 strain (μm).

To study the colony morphology of ES-2 strain on solid media, after 9 days of culture, the colonies from the *M. hyopneumoniae* ES-2 strain on solid medium were observed through microscopic in this study. The colonies were found to be very small and granular with irregular margins with their central parts burrowed into the medium, which exhibited a typical “fried egg” appearance (Figures 2A,B). To further observe the morphology of cells, *M. hyopneumoniae* ES-2 strain was collected by centrifugation. ES-2 strain cells were found to be spherical or elliptic and they differed in sizes under SEM (Figures 2C,D). Meanwhile, TEM results exhibited that the morphology of most cells was spherical and a very small number of them was irregular in morphology (Figures 2E,F). The diameters of ES-2 strain cells ranged from 0.2 to 1.6 μm and the diameters of approximately 80% of cells were between 0.4 and 1.0 μm under the electron microscope (Figure 2G).

### The Relationship Between the Growth Characteristics of Strain ES-2 and Color Shift of Medium

**Figure 3:**
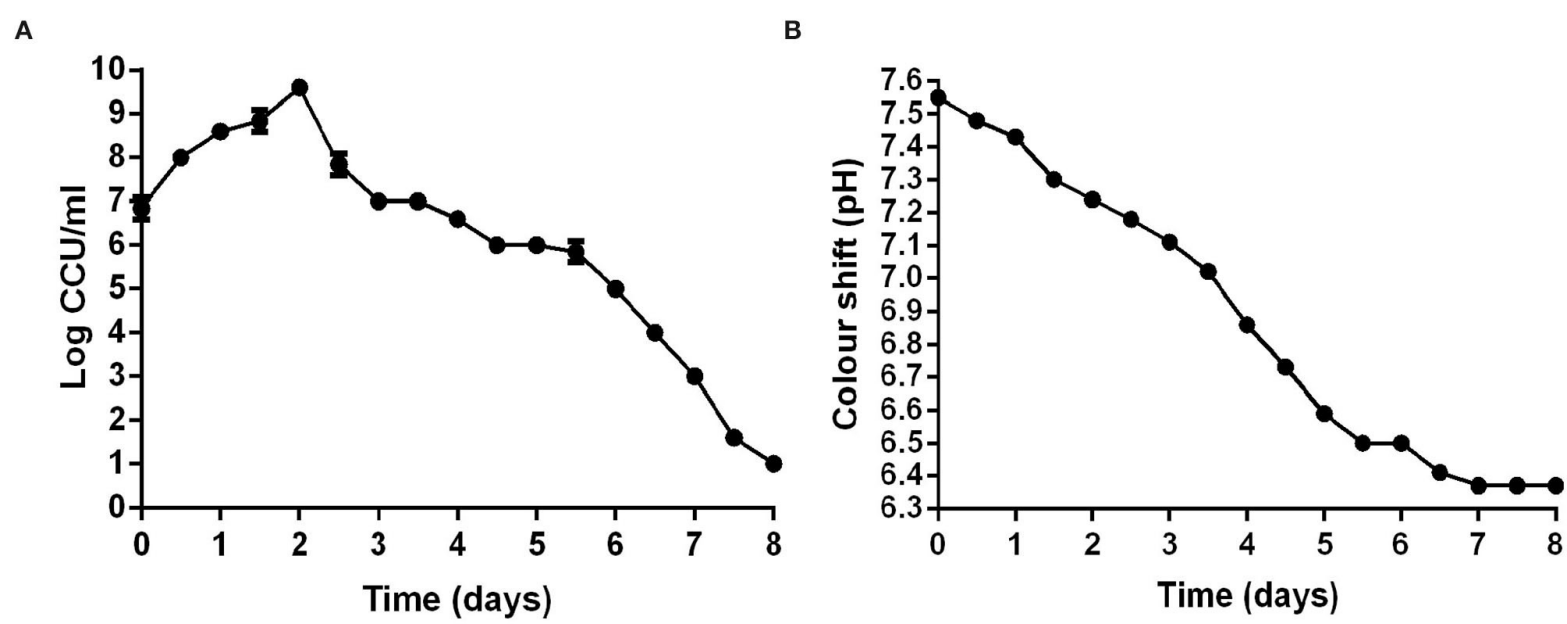
CCU of *M. hyopneumoniae* and pH of corresponding the modified Friis broth from day 0 to day 8. **(A)** CCU of *M. hyopneumoniae* was recorded every 12 h. **(B)** pH of corresponding the modified Friis broth was measured every 12 h. The growth curve of three repeating experiments were developed.

Color-changing units (CCU) of liquid medium were commonly used to determine the viability of *M. hyopneumoniae*. After *M. hyopneumoniae* ES-2 strain culture was transferred into a fresh medium, the results showed that the number of ES-2 strain cells was approximate 10^7^ CCU/ml on day 0 (Figure 3A). From day 0 to day 2, the number of ES-2 strain cells was constantly increasing. However, the pH of the color shift continued to decline (pH 7.55–6.37) during the whole incubation process. And the viability in cultures reached a maximum (10^10^ CCU/ml) approximately on day 2 postincubation with the corresponding pH of 7.24. After 2 days of culture, the viability in cultures continuously declined. On day 8, only 10 CCU/ml of ES-2 strains viability was detected with the corresponding color shift pH being 6.37. On day 7, color shift pH was minimum (6.37) and stopped declining (Figures 3A,B).

### Typical Macroscopic Mycoplasmal Pneumonia Lesions Caused by *M. hyopneumoniae* Strain ES-2

**Figure 4:**
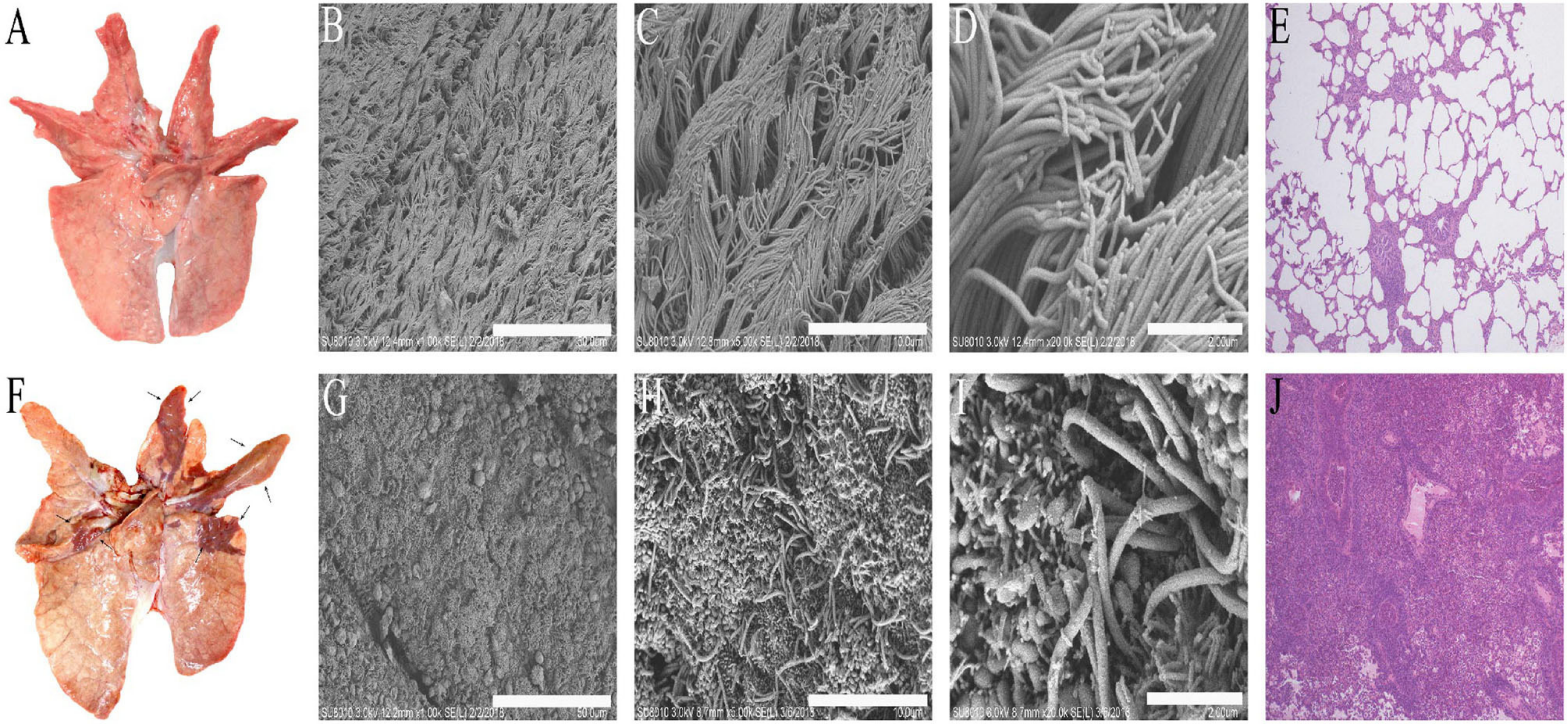
The typical lesions caused by *M. hyopneumoniae* occurred in the lungs of pigs infected with strain ES-2. The lung tissues were observed on day 38 after infection. **(A)** The lung tissues of pigs inoculated with modified Friis liquid medium served as blank group. **(B)** (Bars = 50 μm), **(C)** (Bars = 10 μm), and **(D)** (Bars = 2 μm). The tracheal surface (ciliated epithelial cells and microvilli) was observed by scanning electron microscope. **(E)** (40×) HE staining of lung tissues of blank group is shown. **(F)** Typical macroscopic lesions caused by *M. hyopneumoniae* were observed in the lungs of pigs infected with 7 × 10^7^ CCU of *M. hyopneumoniae* strain ES-2. **(G)** (Bars = 50 μm), **(H)** (Bars = 10 μm), and **(I)** (Bars = 2 μm). The tracheal surfaces of the lungs of pigs infected with strain ES-2 (almost all the fluff is loss) were observed by scanning electron microscope. **(J)** (40×) The hyperplasia of peribronchiolar accumulation of mononuclear cells and the epithelial cells was observed from the lungs of pigs infected with ES-2 by HE staining.

Some studies reported that *M. hyopneumoniae* could infect pigs of various ages resulting in typical lesions of EP (43). In this study, the 28-day-old pigs infected with *M. hyopneumoniae* ES-2 strain for 38 days showed typical pneumonia in the accessory lobes, apical lobes, cardiac lobes, and the cranial portion of the caudal lobes of the lungs (Figure 4F). However, typical lesions of EP were not discovered in the lungs of pigs inoculated with fresh medium without ES-2 strain in the control group (Figures 4A–D). In the pathogenesis of *M. hyopneumoniae* infections, it is crucial for pathogenic *M. hyopneumoniae* to damage the ciliated epithelial cells of the trachea, bronchi, and bronchioles (1, 2). SEM results showed that the cilia of the bronchi in the lungs of the pigs infected with the ES-2 strain were tangled, clumped, split, and lost, compared to those of the healthy lungs (Figures 4G–I). In addition, the histological examination results of the lungs of pigs infected with *M. hyopneumoniae* ES-2 strain showed that much less air was present in the infected lung due to the massive infiltration of inflammatory and lymphocytes cells (Figure 4J), compared to that in the healthy lung (Figure 4E). Therefore, it could be concluded that the *M. hyopneumoniae* ES-2 strain isolate is a pathogenic strain.

### Genomic Characteristics of *M. hyopneumoniae* Strain ES-2

The genomes of 9 *M. hyopneumoniae* strains were available from the National Center for Biotechnology Information (NCBI) database. Of these 9 genomes of *M. hyopneumoniae* strains, the assembly level of 6 genomes (J, 7422, 7448, 168, 168-L, 232, and KM014) was complete. The other 2 genomes (11 and TB1) were incomplete. The genome size of *M. hyopneumoniae* strains ranged from 0.8 to 1.0 Mb (Supplementary Table S1). In this study, the complete genome of *M. hyopneumoniae* ES-2 strain (GenBank accession number GCA_004768725.1), which was sequenced using the PacBio method, contained a 956,514 bp (GC content 28.36%) single circular chromosome. The total number of genes predicted was 733, and the average gene length was 1,164 bp (Supplementary Table S1). Subsequently, 25 virulence factors of *mycoplasma* reported (http://www.mgc.ac.cn/cgi-bin/VFs/compvfs.cgi?Genus=Mycoplasma) were collected from the virulence factor database to investigate the distribution of virulence-related factors among 9 *M. hyopneumoniae* strains. By aligning the proteins (identity ≥ 80% and coverage ≥ 80%) from each strain against the reported 25 virulence factors, a total of 11 virulence factors were identified in these genomes. In total, 10 out of 11 virulence factors were found to be present in each of 9 *M. hyopneumoniae* strains, and some virulence factors had multiple copies in these genomes. The cytoadherence protein (P200) (44) of *Mycoplasma pneumoniae* which functions in cytoadherence and gliding motility was found to be present only in the genome of the ES-2 strain and absent in other strains (Supplementary Table S1). So putative protein P200 has existed in *M. hyopneumoniae* ES-2.

In addition, the above experiments show that *M. hyopneumoniae* strain ES-2 is pathogenic for pigs. Among the *M. hyopneumoniae* strains which have been reported, *M. hyopneumoniae* strains 7422, 7448, 168, 168-L, and 232 were also reported to be pathogenic for pigs. Only *M. hyopneumoniae* strain J was nonpathogenic. The pathogenicity of the remaining 3 strains (KM014, TB1, and 11) was unknown.

### Phylogenetic Relationship Between Different *Mycoplasma* Species

**Figure 5:**
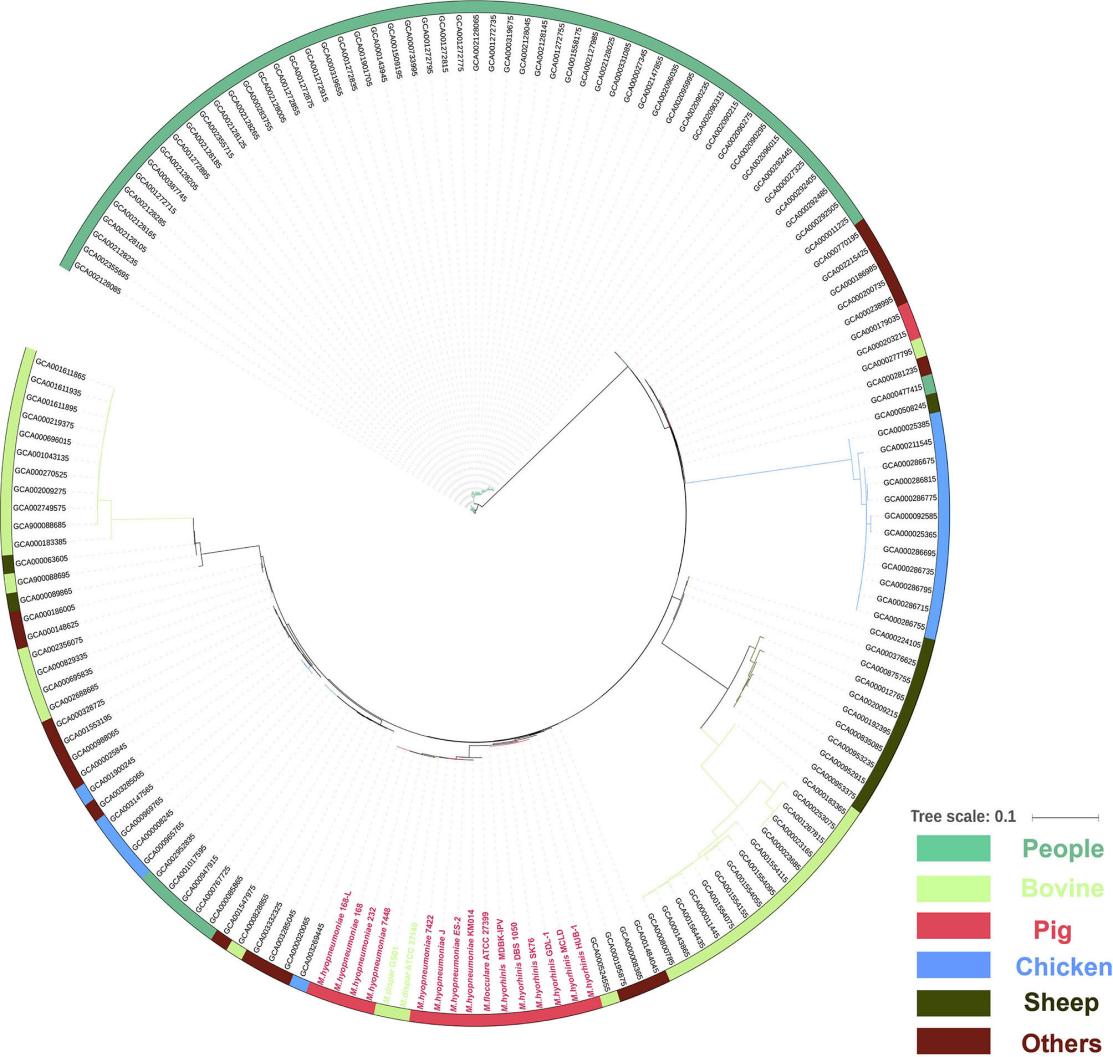
Phylogenetic tree of the whole genome sequences of 162 *mycoplasma* strains isolated from human (green), bovine (light green), pig (red), chicken (blue), sheep (blackish green), and others species (purple) based on amino acid alignments of all genes.

To understand the phylogenetic relationship between different *mycoplasma* species, all 162 complete genomes of different species of *mycoplasma* strains were collected from the NCBI database. However, no core gene was found in the 162 *mycoplasma* strains. Therefore, the phylogenetic tree was first constructed based on CDS existence information in 162 *mycoplasma* strains. The phylogenetic tree showed that 15 porcine *mycoplasmas* (8 *M. hyopneumoniae*, 1 *M. flocculare*, and 6 *M. hyorhinis*), 2 *M. dispar*, 1 *M. bovoculi*, 3 other *mycoplasmas* form a group; 53 human *mycoplasmas* form another group; 10 sheep *mycoplasmas* and 14 bovine *mycoplasmas* form the third group; 12 chicken *mycoplasmas*, 2 human *mycoplasmas*, 2 porcine *mycoplasmas*, 1 bovine *mycoplasma*, and 7 other *mycoplasmas* form the fourth group; and 17 bovine *mycoplasmas*, 6 chicken *mycoplasmas*, 53 human *mycoplasmas*, and 13 other *mycoplasmas* form the fifth group (Figure 5).

### The Genes Present in Pathogenic *M. hyopneumoniae* Strains but Absent in Nonpathogenic *M. hyopneumoniae* Strains

**Figure 6:**
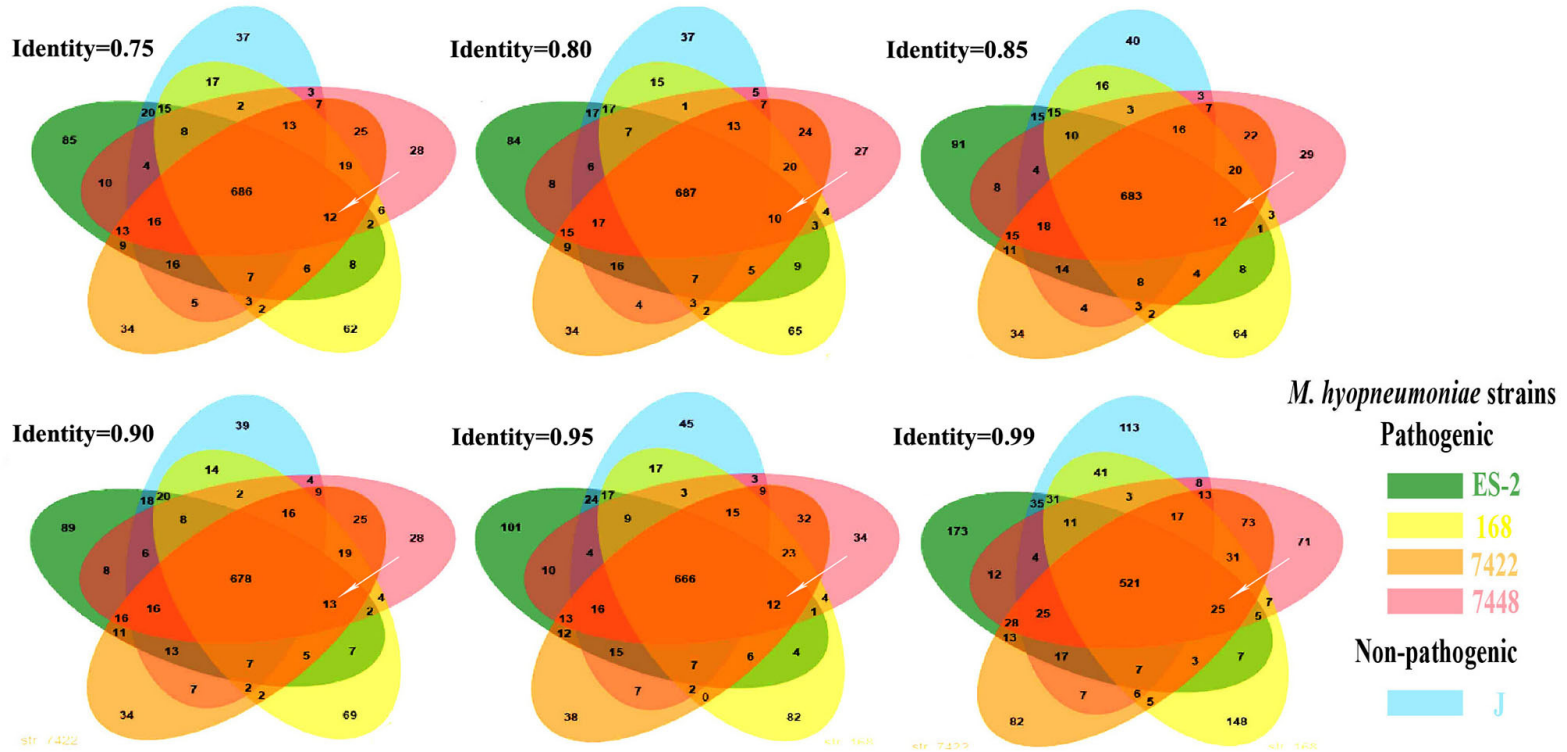
The genes specific to pathogenic *M. hyopneumoniae* strains. These Venn diagrams were developed. After the genes shared by pathogenic *M. hyopneumoniae* (ES-2, 168, 7422, and 7448) strains and nonpathogenic *M. hyopneumoniae* strain J were eliminated based on different levels of identity. 12, 10, 12, 13, 12, and 25 (white arrows) genes specific to pathogenic *M. hyopneumoniae* strains were, respectively, showed at the levels of identity = 0.75, 0.80, 0.85, 0.90, 0.95, and 0.99.

To detect the genes which are present in pathogenic *M. hyopneumoniae* strains but absent in nonpathogenic *M. hyopneumoniae* strains, each gene of 4 pathogenic *M. hyopneumoniae* strains ES-2, 168, 7,448, and 7,422 and each gene of 1 nonpathogenic *M. hyopneumoniae* strain J were aligned based on the different levels of identity (0.75, 0.8, 0.85, 0.9, 0.95, and 0.99) at the amino acid level. Venn diagram of the results showed that the number of common genes shared by 4 pathogenic *M. hyopneumoniae* strains and 1 nonpathogenic *M. hyopneumoniae* strain J were, respectively, 686 (identity = 0.75), 687 (identity = 0.80), 683 (identity = 0.85), 678 (identity = 0.90), 666 (identity = 0.95), and 521 (identity = 0.99). Excluding the common genes shared by pathogenic and nonpathogenic strains, 12 (identity = 0.75), 10 (identity = 0.80), 12 (identity = 0.85), 13 (identity = 0.90), 12 (identity = 0.95), and 25 (identity = 0.99) genes were found to be shared by all 4 pathogenic *M. hyopneumoniae* strains at the amino acid level, respectively (Figure 6). Further analysis of the genes which are present in pathogenic *M. hyopneumoniae* strains but absent in nonpathogenic *M. hyopneumoniae* strains indicated that 34 genes specific to pathogenic *M. hyopneumoniae* strains were detected at the amino acid level, of which 18 were hypothetical proteins, and another 16 were annotated in NCBI database (Table 1). By BlastN, 2 out of 34 genes specific to pathogenic *M. hyopneumoniae* strains at the amino acid level were absent in the nonpathogenic *M. hyopneumoniae* J strain at the nucleotide level (Table 2). ES-2_00484 gene was only identified in pathogenic *M. hyopneumoniae* strains 168, 168-L, 7422, and 7448. A partial sequence of the ES-2_00744 gene was only identified in pathogenic *M. hyopneumoniae* strains 232, 168-L, 7,422, and 7,448. Although the pathogenicity of *M. hyopneumoniae* KM014 is unclear, both genes were identified in the strain.

**Table 1:** The genes present in pathogenic *M. hyopneumoniae* strains but absent in nonpathogenic *M. hyopneumoniae* strains at the amino acid level.

**Specific genes**	**Identity**			**Description**			
	**0.75**	**0.80**	**0.85**	**0.90**	**0.95**	**0.99**	
ES-2_00039	×	×	×	×	×	√	Lipoprotein signal peptidase
ES-2_00047	×	×	×	√	√	√	mtRNA-Pro(tgg)
ES-2_00142	×	×	×	×	×	√	GTPase Der
ES-2_00240	√	√	√	√	√	√	mtRNA-Trp(tca)
ES-2_00267	√	√	√	√	√	×	Phosphopentomutase
ES-2_00285	×	×	×	×	×	√	50S ribosomal protein L17
ES-2_00466	×	×	×	×	×	√	30S ribosomal protein S6
ES-2_00575	×	×	×	×	×	√	Vitamin B12 import ATP-binding protein BtuD
ES-2_00829	√	√	√	√	√	√	mtRNA-Ser(gga)
ES-2_00841	×	×	×	×	×	√	Vitamin B12 import ATP-binding protein BtuD
ES-2_00884	√	√	√	√	√	√	mtRNA-Gln(ttg)
ES-2_01011	×	×	√	×	×	×	mtRNA-His(atg)
ES-2_01096	×	×	×	×	×	√	Vitamin B12 import ATP-binding protein BtuD
ES-2_01145	×	×	×	×	×	√	Methionine aminopeptidase
ES-2_01146	√	√	√	√	√	√	tRNA-His(atg)
ES-2_01167	√	√	√	√	×	×	mtRNA-Ser(cga)
ES-2_00053	×	×	×	×	×	√	Hypothetical proteins
ES-2_00101	×	×	×	×	×	√	Hypothetical proteins
ES-2_00168	×	×	×	×	×	√	Hypothetical proteins
ES-2_00233	×	×	×	×	×	√	Hypothetical proteins
ES-2_00297	×	×	×	×	×	√	Hypothetical proteins
ES-2_00422	×	×	×	×	×	√	Hypothetical proteins
ES-2_00424	×	×	×	×	×	√	Hypothetical proteins
ES-2_00451	×	×	×	×	×	√	Hypothetical proteins
ES-2_00483	√	√	√	√	√	×	Hypothetical proteins
ES-2_00484	√	√	√	√	√	×	Hypothetical proteins
ES-2_00736	√	×	×	√	×	×	Hypothetical proteins
ES-2_00744	√	×	√	×	×	×	Hypothetical proteins
ES-2_00861	×	×	×	×	×	√	Hypothetical proteins
ES-2_00926	√	√	√	√	√	×	Hypothetical proteins
ES-2_00995	√	√	√	√	√	×	Hypothetical proteins
ES-2_01047	×	×	×	×	√	×	Hypothetical proteins
ES-2_01105	×	×	×	×	×	√	Hypothetical proteins
ES-2_01164	×	×	×	×	×	√	Hypothetical proteins

*“×” means that genes was not identified*.

*“√” means that genes was identified*.

**Table 2:** The gene present in pathogenic *M. hyopneumoniae* strains but absent in nonpathogenic *M. hyopneumoniae* strain at the nucleotide level.

**Genes**	***M. hyopneumoniae*** **strains**							
	**ES-2**	**168-L**	**7422**	**7448**	**168**	**KM014**	**232**	**J**
ES-2_00484^a^	+	+	+	+	+	+	-	-
ES-2_00744^b^	+	+	+	+	-	+	+	-

*“+” means that the gene was identified*.

*“−” means that the gene was not identified*.

a *The nucleotide sequence (990bp) of the ES-2_00484 gene is as follows*.

*ATGAATAAAAATAATTATACCACAAAATTTAAAAACACTACTAAAAAAAACAAAAAAAAATTTTGTTCTAATTCGTCTTATATTTTTTACGTGTTTTGAGTAATTAGAGATTTTGAATTT*.

*AAAAAGAAAGGAGAAAAATGTAAGGTTTTAGATGTTAAACAAATTTGAGAGAAGTTAAATGCAAATTGAGAATCTAAAACTTGAAATATAAAAACTATTTATAAAGTTCTAAATTATTTAATTCAA*

*GAGAATATTATTGAAGTACAAAAAATAGCAAATAAATTGATAGTCGTTAATGTTCTGAATTTAAAAAAATATTATATTGCTAAAAAAGTACTTGATTTAAGTATTAATTTAGAAAAATTAGTTTCG*

*GCAAATTACTTAAAAGTTTATCAATATCTAAAAAATTTAGCAATTAAAAATTTCAAATTGAATAACTGAAAAAATAGTAATTATTCACTAGAAAATTCTTATACATATTTCCAAAATTTAGA*

*TTTAAGCAATGAAACTATTGCTAAAGCTCTAAAATGATTTTCAGAAAACAACATTCAAATTGAATTTGAAAATAAAACTATTTCTAAAAAATTAATTTCTAGCGTTAATTTAGACAAAAATACA*

*TTTTTTAAAGGGATTAAATATGAAAAACAATTAATTTCTATCAGTTTAAAAAGCGTTAAAAAAACTTTGTATAGACAGTTTCAAAATAACAAAGCTTATTTTTGGTCAAAAAATTTATTTAAATG*

*AAAAGTTTCAAAAACAATAGTTGTTAAAATCAAAAAATTTTTTGAATATTTGATTAAAAGATCTTGTTATTTGATCAGAAAAACAAAAATTGGGCTTACTTATTTGCCTTGCTGATGCCAAAAT*

*AAGGGAGCCCATTGAAAAATAAATTGCGAAAAACCAAAATATAAATCTTTTTACCAAGAAAAAGATTTGATTATTCGGCGGCAAGGCCAAAAAAATACAAACCTTTTGGTTCTTCAAATTTAA*.

b *The partial nucleotide sequence (369bp) of the ES-2_00744 gene is as follows*.

*TTAGTTAATAATAGATGACACGATAGATGAGATTTTAGCTTATCTAAAAAATTAGAGGCA*.

*ACCGATTATAAATTTATGATTGACTGATTTGAAAAAAATGAATTAAATATCAATATGCAA*.

*GCCTTTAATCAATTTTGCAATGCCATGAAAAAAGAATGACCACAAAGTCATGTTTTTGATTTCGATGAACTCGAATTTCAACTTAAACAAGAAATATCCAAACTAGAAAAACAAAAGCAAATTCA*

*AAATCAAAAGACAATCAAAGATAAATTTATTTATCAAAACGCAAGATTACAAGCTGAAAAATGAGTTCAAGAGGAAGAAGAAAGATTAAAAAAATATGTCCCAAAATTTAAAATGAAAATGTAA*.

## Discussion

At present, Chinese native black pigs are suffering from severe *M. hyopneumoniae* infections. However, little is known about the growth characteristics and pathogenicity of *M. hyopneumoniae* which infects Chinese black pigs, especially Enshi black pigs. To better understand and treat *M. hyopneumoniae* infection in Enshi black pigs, the previously reported methods (7, 19, 37) were used to isolate *M. hyopneumoniae* in the lungs of Enshi black pigs. Unfortunately, due to the inability to completely remove *M. hyorhinis, M. hyopneumoniae* could not be isolated from pathological samples containing the fast-growing *M. hyorhinis* after repeating tests. To effectively remove *M. hyorhinis*, an improved method of multiple pulmonary-point sampling and continuous passage culturing based on previous methods reported (7, 19, 37) was used in this study.

By using the multiple pulmonary-point sampling and continuous passage culturing method, a *M. hyopneumoniae* strain was initially isolated from the lungs of an Enshi black pig (Figure 1). To further purify the *M. hyopneumoniae* strain initially isolated, the *M. hyopneumoniae* strain initially isolated was grown on a solid medium. A single colony was cultured in a liquid medium to obtain the pure *M. hyopneumoniae* strain. After three times of purification, the *M. hyopneumoniae* ES-2 strain was successfully isolated. Subsequently, this study determined that the *M. hyopneumoniae* ES-2 strain was most sensitive to kanamycin (2 μg/ml), which will provide a theoretical basis for the rapid and effective treatment of *M. hyopneumoniae* ES-2 strain infections.

By further study on the growth characteristics of *M. hyopneumoniae* ES-2 strain, we found that the modified Friis medium was most suitable for the growth of strain ES-2. The maximum viability of ES-2 strain in a modified Friis medium reached 10^10^ CCU/ml at approximately 2 days of incubation (Figure 3). Our SEM and TEM exhibited that ES-2 strain cells were round or oval, and the diameters of most cells were between 400 and 1,000 nm (Figure 2), which was consistent with the reports by other researchers (1) that *M. hyopneumoniae* cells were coccoid or coccobacillary, and their mean diameters range from 0.5 to 0.8 μm. Previous studies reported that pathogenic *M. hyopneumoniae* caused *mycoplasma*-like pneumonia lesions in the parts of the cardiac, apical, diaphragmatic, and accessory lobes, and led to the ciliostasis, clumping, and final loss of the cilia on the epithelial surface of the trachea, bronchi, and bronchioles (12). In agreement with previous reports, our findings showed that the lungs of the pigs inoculated with *M. hyopneumoniae* ES-2 strain exhibited the *mycoplasma*-like pneumonia lesions (Figure 4F), the increased mononuclear cells in peribronchiolar (Figure 4J), and the ciliostasis, clumping, and final loss of cilia by SEM (Figures 4G–I). These results revealed that ES-2 is a pathogenic *M. hyopneumoniae* strain. To further discover the virulence factor of *M. hyopneumoniae* ES-2 strain, 25 *mycoplasmas-*virulence-related genes previously reported were aligned with each gene of pathogenic and nonpathogenic *M. hyopneumoniae*. Results showed that 11 *mycoplasmas-*virulence-related genes were found to be present in all reported *M. hyopneumoniae* strains, and that only the P200 gene, which was crucial for *M. pneumoniae* to glide and colonize in the differentiated bronchial epithelium, was discovered to be present in ES-2 strain (Supplementary Table S1). Therefore, we speculate that the putative p200 plays an important role in the pathogenesis of the *M. hyopneumoniae* ES-2 strain.

In general, *M. hyopneumoniae*, as one of the main causes of complex porcine respiratory diseases, which works together with other pathogens (PCV-2, PRRSV, etc.), plays a crucial role in the swine respiratory disease (45, 46). Moreover, not only *M. hyopneumoniae* can cause *Mycoplasma*-like pneumonia lesions (44) but also viruses, particularly, the swine influenza virus may cause similar pneumonia lesions (13, 28). However, there was no effective method to distinguish commensal agents from potential pathogenic agents in pigs suffering from respiratory disease (47). Therefore, it is very implicated and difficult to diagnose and treat porcine respiratory disease caused by multiple pathogens infections. Considering the frequent clinical outbreaks of respiratory diseases, the development of a new method that could not only detect the presence of a pathogen but also identify whether it is pathogenic or nonpathogenic in the infected pig lungs will contribute to the accurate prevention and effective treatment of complex porcine respiratory diseases. Therefore, it is very important to discover the genes specific to pathogenic *M. hyopneumoniae* strains.

In this study, the phylogenetic tree revealed that pathogenic and nonpathogenic *M. hyopneumoniae* strains form a group (Figure 5). Therefore, pathogenic and nonpathogenic *M. hyopneumoniae* strains cannot be distinguished by phylogenetic analysis. To distinguish between pathogenic and nonpathogenic *M. hyopneumoniae* strains, this study explores the genes specific to pathogenic *M. hyopneumoniae* strains by comparative genomics. In previous studies, there were only several *M. hyopneumoniae* strains reported, and their complete genome sequences (24, 31–33) have been available in the GenBank. In these reported *M. hyopneumoniae*, only the J strain was nonpathogenic, which was often considered the reference strain (23, 48, 49). Although the J strain was a highly passaged strain that is incapable of colonizing pigs, the strain as a nonpathogenic *M. hyopneumoniae* was often compared with pathogenic *M. hyopneumoniae* strains in comparative genomics (23, 24). In the present study, the pathogenic *M. hyopneumoniae* ES-2 strain was isolated and its complete genome was sequenced by the PacBio method (Supplementary Table S1). Venn diagram developed at different levels of identity showed that 34 genes specific to pathogenic *M. hyopneumoniae* strains were found (Figure 6 and Table 1) at the amino acid level. Further analysis identified that 2 genes are present in pathogenic *M. hyopneumoniae* strains but absent in nonpathogenic *M. hyopneumoniae* strains based on amino acid and nucleotide level indicated (Table 2).

In summary, this study successfully isolated and purified a pathogenic *M. hyopneumoniae* ES-2 strain from the lungs of Enshi black pigs, which will help us quickly determine the susceptibility of *M. hyopneumoniae* ES-2 strain to drugs, and guide the clinical rapid and accurate drug selection. In addition, the phylogenetic tree revealed that there may be a selective preference in genome grouping and natural recombination among different *Mycoplasma* species. These results may develop the foundation for *Mycoplasma* grouping in the future. Further research results identified that 2 genes are present in pathogenic *M. hyopneumoniae* strains but absent in nonpathogenic *M. hyopneumoniae* strains at the amino acid and nucleotide level, which may be predicted to be used as a molecular marker to determine whether *M. hyopneumoniae* strains are pathogenic or nonpathogenic in clinical diagnosis. Therefore, it is meaningful and important to prevent and treat *M. hyopneumoniae* infection in Chinese black pigs by studying the characteristics of *M. hyopneumoniae* strain ES-2.

## Data Availability Statement

The datasets presented in this study can be found in online repositories. The names of the repository/repositories and accession number(s) can be found in the article/Supplementary Material.

## Ethics Statement

The animal study was reviewed and approved by Animal Care and Use Committee of Wuhan Polytechnic University (No: WPU202201002).

## Author Contributions

YZha, BZ, HC, and CT contributed to the conception and design of the study. YZhu organized the database. YZha, BZ, and YZhu performed the statistical analysis. YZha, BZ, and CT wrote the first draft of the manuscript. ML and XW wrote sections of the manuscript. All authors contributed to manuscript revision, read, and approved the submitted version.

## Funding

This work was supported by grants from the National Natural Science Foundation of China (32102674), the National Key R&D Program of China (2017YFD0500202), the Hubei Province Natural Science Foundation for Innovative Research Groups (2016CFA015), the earmarked fund for China Agriculture Research System (CARS-35), and the Hubei Agricultural Science Innovation Centre (2016-620- 000-001-039).

## Conflict of Interest

The authors declare that the research was conducted in the absence of any commercial or financial relationships that could be construed as a potential conflict of interest.

## Publisher's Note

All claims expressed in this article are solely those of the authors and do not necessarily represent those of their affiliated organizations, or those of the publisher, the editors and the reviewers. Any product that may be evaluated in this article, or claim that may be made by its manufacturer, is not guaranteed or endorsed by the publisher.
