# Supplementary material for: Characteristics of Mycoplasma hyopneumoniae Strain ES-2 Isolated From Chinese Native Black Pig Lungs
Source: Front Vet Sci. 2022 Jun 30;9:883416. doi: 10.3389/fvets.2022.883416 (PMC9280346; doi:10.3389/fvets.2022.883416)
Supplement: Supplementary file 1 [file Table_1.DOC]

**Table 1. Comparison of general features of different *M.*** ***hyopneumoniae* strains and the presence of known *mycoplasma***

**virulence factors in different *M.* *hyopneumoniae* strains**

|  | *Mycoplasma hyopneumoniae* | | | | | | | | |  |
| --- | --- | --- | --- | --- | --- | --- | --- | --- | --- | --- |
|  | ES-2 | 168 | J | 7422 | 7448 | 232 | KM014 | TB1 | 11 | |
| Assembly level | Complete Genome | Complete Genome | Complete Genome | Complete Genome | Complete Genome | Complete Genome | Complete Genome | Scaffold | Scaffold | |
| Total length (bp) | 956,514 | 925576 | 897,405 | 898495 | 920079 | 892758 | 964503 | 909064 | 898877 | |
| G+C content % | 28.36 | 28.4 | 28 | 28.4 | 28.5 | 28.6 | 28.73 |  |  | |
| Total no. of CDSs | 733 | 695 | 679 | 692 | 716 | 692 | 680 |  |  | |
| Average CDS length (bp) | 1164 | 1071 | 1178 | 1147 | 1146 | 1164 | 921 |  |  | |
| No. of rRNAs | 3 | 3 | 3 | 3 | 3 | 3 | 3 | 3 | 3 | |
| No. of tRNAs | 30 | 30 | 30 | 30 | 30 | 30 | 32 | 30 | 30 | |
| Known virulence factors of *Mycoplasma* |  |  |  |  |  |  |  |  |  | |
| Elongation factor Tu | 4^a^ | 4 | 4 | 4 | 4 | 4 | 4 | 3 | 4 | |
| Lipoprotein T, P102 paralog, surface adhesion | 1 | 1 | 1 | 1 | 1 | 1 | 2 | 1 | 1 | |
| Adhesin like-protein P146 | 3 | 2 | 2 | 3 | 2 | 3 | 2 | 1 | 3 | |
| P76 membrane protein precursor | 1 | 1 | 1 | 1 | 1 | 1 | 2 | 1 | 1 | |
| Cyto adherence proteins | 1 | 0 | 0 | 0 | 0 | 0 | 0 | 0 | 0 | |
| Putative p216 surface protein | 1 | 1 | 1 | 1 | 1 | 1 | 1 | 1 | 1 | |
| Prolipoprotein p65 | 3 | 3 | 3 | 3 | 3 | 3 | 4 | 2 | 3 | |
| Pyruvate dehydrogenase | 1 | 1 | 1 | 1 | 1 | 1 | 1 | 1 | 1 | |
| Haemolysin | 1 | 1 | 1 | 1 | 1 | 1 | 1 | 1 | 1 | |
| P48, predicted lipoprotein | 1 | 1 | 1 | 1 | 1 | 1 | 1 | 1 | 1 | |
| Membrane nuclease, lipoprotein | 1 | 1 | 1 | 1 | 1 | 1 | 1 | 1 | 1 | |

a The number of copies of genes in genome.
